# Advanced Studies on Toxic Chemicals: Properties and Characteristics

**DOI:** 10.3390/toxics10080475

**Published:** 2022-08-15

**Authors:** Miguel A. Esteso

**Affiliations:** 1 Universidad Católica de Ávila, Calle los Canteros s/n, 05005 Ávila, Spain; mangel.esteso@ucavila.es; 2 Universidad de Alcalá, U.D. Química Física, 28805 Alcalá de Henares, Spain

Examining the toxic scope of substances used in daily life (referred to as Contaminants of Emerging Concern (CEC)) in food, medicines, utensils, work and other industries, provides us with interesting information that will help us to prevent and recover from the dangerous organic unbalances that these substances can cause. The increase in the presence of these pollutants in domestic wastewater is already cause for concern; on one hand, it is indicative of the parallel increase in such pollutants in our daily lives; on the other hand, it poses a significant risk to ecosystems, since domestic WWTPs are generally ill-prepared to eliminate this type of substances.

Many of these substances originate from the manufacturing industry; for example, there are a growing number of studies related to the toxicological effect of certain nanomaterials used in the field of biomedicine. Likewise, the growing presence in WWTPs of heavy metal ions, as well as surfactant substances, poses a significant risk to both human health and the ecosystem due to its well-known adverse health effects.

The ongoing COVID-19 pandemic has brought the use of disinfectants (antiviral and antibacterial) to the forefront. We have incorporated it into our daily routine without having had time to carry out a rigorous study of the potential toxic effects that these disinfectants can produce on both people and the environment, once eliminated into wastewater.

In summary, studies related to the properties and characteristics of this type of chemical toxics (CEC) are both necessary and important. By improving our knowledge about them, we will be able to control the presence of the contaminants with which we share everyday life and, above all, to resolve or at least mitigate their damaging effects.

This Special Issue (SI) includes two papers [1,2] focused on removing highly toxic heavy metal cations (Pb^2+^, Hg^2+^, Cu^2+^ and Cd^2+^) by complexing them with water-soluble resorcinarenes, both simple and modified, which have turned out to be highly selective for these ions. Castillo-Aguirre et al. [1] used a copolymer of poly(BuMA–*co*–EDMA) modified, by means of the impregnation method, with C-tetra(nonyl)calix[4]resorcinarene for the removal of highly toxic cations (Pb^2+^, Hg^2+^, and Cd^2+^) from aqueous solutions. These authors found that this modified copolymer can be used during several adsorption–desorption cycles without a significant loss of adsorption capacity, the best results being those obtained for the extraction of Pb^2+^, using the resorcinarene for various cycles (for different pH, time of contact and volume of sample) without a significant reduction in adsorption capacity. On the other hand, Sanabria et al. [2] used two water-soluble resorcin[4]arenes to form complexes with these ions (Pb^2+^, Hg^2+^, Cu^2+^ and Cd^2+^). They found that selective complexation with Cu^2+^ y Pb^2+^ was produced by one of the resorcinarenes used, and complexation with Hg^2+^ was produced by the other.

Polychlorinated biphenyls (PCBs), generally present in oils used as insulators, are also important environmental pollutants. An article included in this SI [3] focused on establishing the optimal conditions in which a photocatalytic oxidation technique can be used, together with biotreatment using *Nostoc* sp. microorganisms, to degrade the PCBs present in used dielectric oils. The authors of this paper found that an adequate adjustment of the pH value, as well as of the concentrations of both PCB and titanium dioxide, allows us to reach yields up to 90% in the degradation of PCBs. On the other hand, in relation to the use of the *Nostoc* sp., the authors of this paper found that the presence of these PCBs substances caused an alteration in both the density and growth rate of said microorganisms, probably as a consequence of the toxic effect of those substances on the *Nostoc* sp.

Attention has also been directed towards widely used drugs that are generally present in wastewater as polluting substances. Thus, a paper included in the SI was dedicated to the study of 5-fluorouracil (5-FU) [4], a drug widely used in the treatment of several types of cancer and thus is quite prevalent in hospital wastewater. Its high toxicity is a serious risk to aquifers. The paper shows that 5-FU is successfully encapsulated by both *β*-cyclodextrin (*β*-CD) and sodium dodecyl sulfate (SDS) (to a greater extent by SDS, indicating that SDS is more efficiently encapsulates the 5-FU drug), a characteristic that can be used in its removal from waste solutions. The authors carried out this study on the basis of experimental measurements of the ternary diffusion coefficients obtained by the Taylor dispersion technique, verifying that the cross-diffusion coefficient values of 5-FU with both *β*-CD and SDS were nonzero, in relation to the presence of complex species present in the medium.

Another article in this SI is devoted to the study of carbamazepine, ibuprofen, triclosan and sulfamethoxazole [5], which are widely used in pharmacology, and the various procedures used to control and eliminate these drugs from wastewater through the use of various complexing agents. Díaz-Cubilla et al. show that the presence of these drugs in wastewaters, where they remain untreated in the liquid phase and adhere to the biomass of the anaerobic batch reactors used in the wastewater treatment plants (WWTP), alters normal functioning. This has a considerable negative impact on the anaerobic digestion process, since it reduces the organic matter removal into wastewater, produces microbial stress, cell damage and even causes cell death. The authors concluded that more studies are necessary to improve our understanding of the complex relationships between the trophic groups in anaerobic reactors.

Another work included in this SI deals with the possible sensitization induced by polyethylene glycol (a polymer widely used for surface modification in the pharmaceutical industry and in the field of biopharmaceuticals) on skin tissue [6], e.g., an immune response. The authors concluded that such PEG coatings do not induce an immune response in the skin tissue.

Moreover, this SI includes works related to the improvement of techniques for the recognition and analysis of substances that are toxic to humans. Thus, we collected works related to the development of highly selective analytical methods, for forensic purposes, using different instrumental techniques (liquid chromatography coupled with high resolution mass spectrometry, and Raman microscopy and OPLS-DA (Orthogonal Partial Least-Squares Discriminant Analysis)). Its use allows the determination of new psychoactive substances (such as 2C-B) at ultratrace concentration in a capillary matrix [7], and allows us to distinguish different varieties of marijuana [8] by identifying the most significant spectral Raman bands (obtained using a chemometrics-assisted method based on Raman microscopy and OPLS-DA and supported by quantum chemical simulated Raman spectra) that are useful for the characterization of different marijuana varieties. It also enables detection of anionic surfactants (whose growing presence in aquatic environments, as a consequence of their extensive use in many industries, as well as in households, represents a significant threat due to their high toxicity for aquatic organisms) in alternative solvents to chloroform [9] which have a higher extraction efficiency toward these anionic surfactants.

Finally, a work related to a new type of chlorine dioxide (ClO_2_) gas generator (Dr. CLO^TM^ (NON Inc., Geumsan, Korea)) is included. It can be used to provide a safe indoor environment due to its technology, which stabilizes both the concentration and the release of ClO_2_ gas [10]. On that technical basis, it could suppress viral amplification and prevent viral infections.
